# Catching Takayasu Early: Diagnosing the “Pulseless” Disease in a Child with Palpable Pulses

**DOI:** 10.1155/2021/8885944

**Published:** 2021-05-27

**Authors:** A. R. Santosh Rao, Vinay Jahagirdar, Kaanthi Rama

**Affiliations:** ^1^Amrutha Children's Hospital, Shamshabad, Hyderabad 501218, India; ^2^Gandhi Medical College and Hospital, Secunderabad 500003, India

## Abstract

Takayasu arteritis is a systemic vasculitis of large vessels that mainly involves the aorta and its branches. It normally presents in the third decade of life and is rarely seen in children. We report a case of childhood Takayasu arteritis, in a 12-year-old girl, who presented with abdominal pain and hypertension. Although all her peripheral pulses were palpable, there was a discrepancy between her upper and lower limbs' blood pressure. CT angiography revealed stenosis of the abdominal aorta, at the origin of the celiac artery and right renal artery. She was started on steroids and an antihypertensive, after which she attained remission. Five years down the line, the child has sustained remission, with no signs of disease progression. Early diagnosis of Takayasu and initiation of immunosuppression, before the onset of the classic “pulseless” phase, may contribute to improved long-term prognosis.

## 1. Introduction

Takayasu arteritis (TAK) is a large-vessel vasculitis that mainly affects the aorta and its primary branches. Japanese ophthalmologist, Mikito Takayasu, first described the condition in 1908, in a young female with retinal changes. Later in 1928, Shimzu and Sano described it as a “pulseless” disease.

TAK is distributed worldwide, with the greatest prevalence in Asia [1]. It most commonly affects women in their thirties [2]. Childhood Takayasu arteritis (c-TA) is rarely identified and reported, due to its nonspecific and varied presentations.

The European League Against Rheumatism/Pediatric Rheumatology International Trials Organization/Pediatrics Rheumatology European Society (EULAR/PRINTO/PRES) proposed validated criteria for the diagnosis of c-TA, included in Table 1 [3].

Immunosuppressants remain the mainstay of treating c-TA. Corticosteroids are generally the initial drugs of choice. Methotrexate and mycophenolate may be used down the line to achieve and maintain remission. Surgical revascularization improves the quality of life in case of severe stenosis.

We report a case of an adolescent girl diagnosed with c-TA, early in the course of the disease, before the disappearance of pulses.

## 2. Case Presentation

A 12-year-old girl was brought to the clinic by her parents for pain in her abdomen for a month. She described a constant, dull-aching type of pain in her epigastric region that was nonradiating and increasing with food. There were no relieving factors. The child had a reduced appetite. She denied fever or pain during micturition. She had not attained menarche at the time of the initial presentation.

On examination, the child seemed cachectic. Her height was 142 cm and weight 34 kg, with a body mass index (BMI) of 17. She was afebrile, with a pulse rate of 78/minute and a respiratory rate of 28/minute. Blood pressure was not recorded. Cardiac auscultation revealed S1 and S2 within normal limits. There were no murmurs, rubs, or gallops. Respiratory auscultation showed bilateral air entry and normal vesicular breath sounds, with no added sounds. The abdomen was soft to palpate, with no organomegaly. Suspecting acute gastritis, she was started on a two-week course of proton pump inhibitors.

Ten days later, the child was brought back to the clinic, as there was no respite in her abdominal pain. On examination, the pulse rate was 80/minute, normal in volume and character. All peripheral pulses were equal, with no radiofemoral or radioradial delay. Her respiratory rate was 28/minute. Blood pressure in her right upper limb was 130/85 mm Hg and in her right lower limb was 110/75 mm Hg (95^th^ percentile BP for a 12-year-old female child with height 142.5 cm being 118/78 mm Hg) [4]. The abdomen was soft to palpate, with no organomegaly. There was no lymphadenopathy. Abdominal auscultation revealed a bruit. Neurological examination, visual acuity, and fundoscopic eye examination were normal. A gynecological consult ruled out gynecological issues.

Urine examination showed a red blood cell count of 15–20/HPF. Urine had no albumin or pus cells, ruling out a urinary tract infection. Negative urine ketone bodies and random blood sugar of 102 mg/dl ruled out diabetic ketoacidosis. Hemoglobin was 10 g/dl. Red blood cells, white blood cells, and platelet counts were within normal limits. Serum creatinine, serum albumin, and spot urine albumin-to-creatinine ratio were normal. Erythrocyte sedimentation rate (ESR) was elevated, with ESR after an hour measuring 56 mm/h and after the 2^nd^ hour being 110 mm/hr. C-reactive protein was raised to 24.0 mg/dl. Serum creatinine was 1.0 mg/dl. Transthoracic echocardiogram showed no abnormality in the heart chambers, valves, septae, systemic, and pulmonary vasculature, with pressures and volumes within normal limits. Rheumatoid factor, antinuclear factor, anti-double-stranded DNA antibodies, and antineutrophil cytoplasmic antibodies were negative. Complement factors C3 and C4 were normal. Hepatitis B surface antigen was negative.

**Figure 1:**
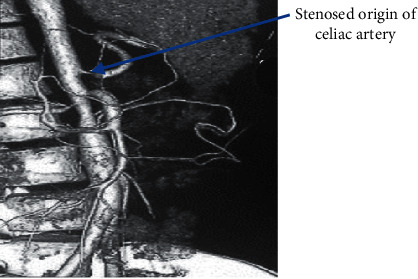
CTA showing the stenosed origin of the celiac artery.

**Figure 2:**
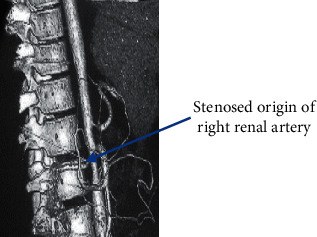
CTA showing the stenosed origin of the right renal artery.

**Figure 3:**
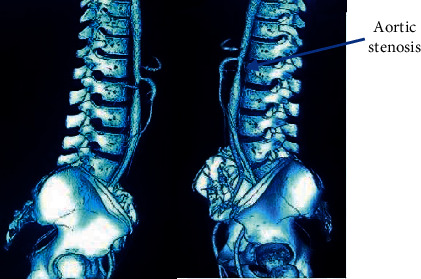
CTA showing aortic stenosis at L2 level.

**Figure 4:**
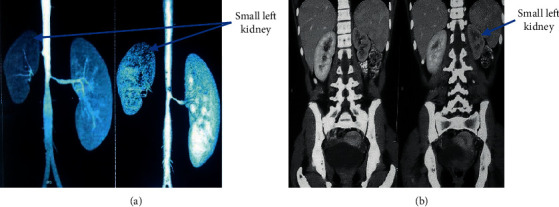
: CTA showing small left kidney.

The clinical presentation of the patient—her adolescent age group, female sex, hypertension, and cachexia—led to suspicion of a multisystem involvement, probably a connective tissue disorder. A CT angiography (CTA) ordered to rule out large-vessel vasculitis showed stenosis at the origin of the celiac artery (Figure 1), at the origin of the right renal artery (Figure 2), and aortic stenosis at the L2 level (Figure 3). Postprandial epigastric pain could be attributed to celiac artery stenosis, and her aortic stenosis could be causing the abdominal bruit. Fluorodeoxyglucose positron emission tomography (FDG-PET) revealed increased FDG uptake, with aortic medial thickening at the level of stenosis. A radioisotope renogram, using diethylenetriaminepentaacetic acid (DTPA), showed severely impaired parenchymal tracer uptake in the left kidney, with prolonged intrarenal transit time and clearance, indicating severely impaired functioning of the left kidney. Right kidney functioning was good, with nonobstructive clearance (Figures 4(a) and 4(b)).

The child was diagnosed with childhood Takayasu arteritis, as per the EULAR/PRINTO/PRES criteria (Table 1). She was started on a course of prednisone 2 mg/kg body weight. Amlodipine 5 mg was initiated for hypertension. Regular follow-up visits were scheduled once in 3 months, where blood pressure, CRP, and serum creatinine were monitored. Prednisone was eventually tapered within 3 months, to 10 mg once a day.

Five years down the line, the patient is doing well, with scheduled follow-ups, once in 6 months. She is healthy, with her weight at 55 kg, height 154 cm, and BMI of 23. All peripheral pulses are felt, equal in volume and character. She is off antihypertensives, and her blood pressure is appropriate for age, sex, and height, without any discrepancy between upper and lower limbs. There is no RF or RR delay, or abdominal bruit. Her CRP levels and urine analysis that are repeated periodically are normal. The patient remains on a low dose of steroids (10 mg prednisone). There are no signs and symptoms suggestive of steroid toxicity. She attained menarche and has regular periods. She is doing well academically, with regular school attendance. Echocardiogram shows no signs of disease progression, with a healthy heart and vasculature, and normal pressures and volumes. She has not needed a surgical revascularization procedure to date.

## 3. Discussion

Takayasu's arteritis (TA) is a chronic granulomatous vasculitis of unknown etiology that involves the aorta and its branches, producing vascular sequelae with stenotic lesions and/or thrombus formation.

The incidence of TA in adults is 1/million/year in Europe and 2.6/million/year in North America [1, 5]. As reported in patients from all ethnicities, the disease is more frequent amongst Asians [6]. TA has a female preponderance, with a female-to-male ratio of 8.5 : 1 [7]. It is more common in the second and third decades of life [8]. However, the exact incidence of c-TA is unknown. In a study by Ruchika et al., the median age of onset was 12.5 years, amongst 40 children with c-TA [9].

The most frequent presentation of c-TA is hypertension (82.6%), followed by headache (31%), fever (29%), dyspnea (23%), weight loss (22%), vomiting (20.1%), and abdominal pain (16.6%) [10]. Due to the nonspecific nature of the symptoms, diagnosing c-TA can be challenging.

There is no specific laboratory marker for TA. 53% of c-TA patients have elevated ESR, and ESR can be used as a lab indicator for disease activity [9]. CRP can also be used as a marker. Novel TA markers include tissue plasminogen activator, intercellular adhesion molecule-1, vascular cell adhesion molecule-1, E-selectin, and platelet endothelial cell adhesion molecule-1 [11].

Vascular imaging for c-TA can be done by conventional, magnetic resonance angiography (MRA), or CT angiography (CTA). Doppler ultrasound can be complementary. Vessel walls can be studied using gadolinium-enhanced MRA or FDG-PET, which shows increased glucose metabolism in inflammatory cells and may be used as a screening method [12]. Thoracic and abdominal aortas are the most commonly involved vessels, with stenosis being the most common lesion seen [11]. Apart from vessel wall abnormalities, evidence of increased collateral circulation can also be seen on angiography [13]. Collaterals indicate the chronicity of stenotic lesions and can delay the need for surgical revascularization [13, 14]. Axial T_1_-weighted MRA shows abnormal wall thickening of vessels and is preferred over CTA in view of exposure to radiation and iodinated contrast dye. Arterial tissue biopsy, if available, shows active inflammatory infiltrate, with lymphocyte predominance [15]. Granulomatous inflammation with giant cells is seen in the media [16].

Due to overlapping clinical presentations, other diagnoses have to be ruled out while considering c-TA. The differential diagnosis includes developmental disorders (aortic coarctation and Marfan syndrome), autoimmune disorders (primary vasculitides such as Behçet's disease and Kawasaki disease, and secondary vasculitides such as SLE and sarcoid), or infectious aortitis (tuberculosis and syphilis). Hypertension, along with blood pressure discrepancy in limbs, may warrant evaluation for aortic coarctation (CoA), thrombus in the descending aorta, or midaortic syndrome.

Aortic coarctation results from obstruction to blood flow in the aorta and most commonly occurs distal to the left subclavian artery, where the ductus arteriosus connects to the aorta. Adolescents with CoA present with headaches due to hypertension and lower limb claudication due to chronic hypoperfusion. Clinical signs include upper extremity hypertension, arm-leg blood pressure gradient, weak femoral pulse, and a systolic murmur on the back due to turbulent flow through the narrowed segment, or a continuous murmur due to collateral flow around the coarctation site. TTE, MR, or CT angiography can be used to identify the aortic arch anatomy. Surgical repair, transcatheter balloon angioplasty, and transcatheter stent implantation are the available treatment modalities [17].

Marfan syndrome may present with aortic coarctation, though aortic dissection is more frequently reported in Marfan [17, 18]. However, it classically encompasses other musculoskeletal (reduced upper segment/lower segment and increased arm span/height) and ocular abnormalities (ectopia lentis).

Fibromuscular dysplasia, which also presents with hypertension, is an important differential but is generally seen in older females, with a median age of presentation around 48 years [19].

BP discrepancy, coupled with a normal aortic arch and proximal aorta, may hint towards thoracic aortic mural thrombus (TAMT) of the descending aorta. Rarely seen in the healthy, the thrombus may be formed due to aortic dissection, aortic aneurysm, malignancy, or blood disorders. Depending on the location of the thrombus, distal embolization may lead to stroke, mesenteric ischemia, organ dysfunction, or acute limb ischemia. CT or MRI angiography can be used to determine the location and extent of the thrombi. Long-term anticoagulation or endovascular surgical options may be used to manage TAMT [20].

Midaortic syndrome is a rare entity, characterized by segmental narrowing of the abdominal or descending thoracic aorta, along with ostial stenosis of the aortic branches. It presents in children with renovascular hypertension, owing to renal artery stenosis. However, unlike c-TA, hypertension is refractory to medical management, and often surgery remains the treatment of choice [21].

The 2018 update of the EULAR recommendations for the management of large-vessel vasculitis recommended that large-vessel vasculitis should be confirmed by imaging or histology. High-dose glucocorticoid therapy (40–60 mg/day prednisone equivalent) must be started immediately for induction of remission in active giant cell arteritis or TAK. A combination of glucocorticoids and nonbiological glucocorticoid-sparing agents should be used in all patients with TAK. Routine antiplatelet or anticoagulant therapy is no longer recommended [22].

Glucocorticoids are effective for the initial treatment of c-TA. Immunosuppressants such as methotrexate and azathioprine can be used to allow the use of a lower dose of steroids, or for maintaining disease control. Alternatives include mycophenolate and leflunomide. Anti-tumor necrosis factor (TNF) agents are used in patients with TA that are not controlled by steroids or immunosuppressants [23]. MRA or CTA can be obtained at the time of diagnosis and may be repeated annually for monitoring disease progression. Even with treatment, around 23% of patients never achieve remission [24]. Endovascular revascularization procedures such as percutaneous transluminal angioplasty (PCTA) may be employed for palliation. Though anti-inflammatory treatment can lead to a dramatic improvement in c-TA, 5-year mortality can be as high as 35% in children [25].

Postprandial pain and weight loss in the setting of a normal abdominal examination can be a vital clue to suspect and diagnose large-vessel vasculitis. Abdominal pain is a common presenting complaint in pediatric practice, and a thorough examination, including palpation of peripheral pulses, auscultation for bruits, and measuring blood pressure in all four limbs, can help to identify a rare clinical entity like c-TA. Through meticulous history, physical examination, and basic tests, we can pick up such multisystemic diseases in their early phase, which may contribute to an improved prognosis. Classically, TA is described as the “pulseless disease,” but in our case, we were able to suspect and confirm it early, before the pulses disappeared. Timely intervention and initiation of treatment prevented the disease from progressing into the “pulseless phase,” signifying the importance of early therapy, in impacting vascular disease activity.

Backed with their medical knowledge, pediatricians must have a high index of suspicion and a low threshold for diagnostic evaluation in any young patient presenting with unexplained hypertension and postprandial pain. Owing to the progressive and fatal nature of c-TA, early diagnosis is crucial to start immunosuppressive therapy and improve outcomes.

## Figures and Tables

**Table 1:** Proposed classification criteria for childhood Takayasu arteritis.

Angiographic abnormalities (conventional, CT, or magnetic resonance angiography) of the aorta or its main branches and at least one of the following criteria	
1	Decreased peripheral artery pulses and/or claudication of extremities
2	Blood pressure difference between arms or legs of >10 mm Hg
3	Bruits over the aorta and/or its major branches
4	Hypertension (defined by childhood normative data)
5	Elevated acute phase reactant (erythrocyte sedimentation rate or C-reactive protein)

## Data Availability

The literature review data used to support the findings of this study are included within the article.
